# High Trait Attention Promotes Resilience and Reduces Binge Drinking Among College Students With a Family History of Alcohol Use Disorder

**DOI:** 10.3389/fpsyt.2021.672863

**Published:** 2021-05-13

**Authors:** Amanda Elton, J. Hunter Allen, Mya Yorke, Farhan Khan, Qiaosen Lin, Charlotte A. Boettiger

**Affiliations:** ^1^Department of Psychology and Neuroscience, University of North Carolina at Chapel Hill, Chapel Hill, NC, United States; ^2^Bowles Center for Alcohol Studies, University of North Carolina at Chapel Hill, Chapel Hill, NC, United States; ^3^Biomedical Research Imaging Center, University of North Carolina at Chapel Hill, Chapel Hill, NC, United States; ^4^Brody School of Medicine, East Carolina University, Greenville, NC, United States

**Keywords:** resilience, attention, impulsivity, family history, alcohol use disorder, binge drinking, addiction

## Abstract

Binge patterns of alcohol use among post-high school emerging adults are associated with both immediate negative consequences and increased risk of long-term drinking problems, particularly among individuals with a family history (FH) of alcohol use disorder (AUD). Therefore, the developmental time period of emerging adulthood, paired with the high-risk environment of college campuses, represents an important target for interventions. Attentional ability has recently emerged as a mediator of resilience to stress-related psychopathology and offers a potential neurocognitive target for interventions. We tested the hypothesis that attentional ability promotes resilience to binge drinking in a sample of 464 college students with (*n* = 221) or without (*n* = 243) familial risk for AUD. Two-way analyses of covariance (ANCOVA) tested effects of FH and self-reported binge drinking on attention scores from the Barratt Impulsiveness Scale (BIS). In addition, mediation analyses tested whether BIS attention scores mediated the relationship between Conner-Davidson Resilience Scale scores and binge drinking. ANCOVA results indicated a significant FH-by-binge drinking interaction (*p* = 0.008) in which FH positive subjects who did not binge drink had the fewest attention problems, consistent with a marker of resilience. Furthermore, BIS attention scores significantly mediated the effect of Conner-Davidson Resilience Scale scores on binge drinking, with stronger effects in FH positive subjects (*p* < 0.001) than FH negative subjects (*p* = 0.49). The findings suggest that attention promotes resilience to binge drinking in individuals with familial risk for AUD. Interventions targeting attentional ability in this high-risk population, particularly FH positive individuals with attention deficits, may serve to reduce binge drinking and its consequences.

## Introduction

Approximately 7% of adults in the United States meet criteria for an AUD, with the highest prevalence among individuals between late adolescence and young adulthood (1, 2). In fact, most first-time cases of AUD occur in emerging adulthood, around 18–20 years of age (3), and approximately one third of US college students meet criteria for an AUD (4). College campuses provide a unique environment that particularly promotes participation in hazardous drinking such that college students engage in heavier drinking (5) and exhibit greater increases in drinking levels following high school than their non-college-attending peers (6). Moreover, college attendance enhances effects of genetic risk on alcohol consumption (7).

Binge drinking in particular represents a dominant pattern of drinking among college students, with 39% of these individuals (aged 18–22 years) reporting at least one binge drinking episode in the past month (2). This pattern of drinking is not only associated with later drinking problems (8) but is also directly linked to negative consequences including alcohol poisoning, accidental injuries and deaths, physical and sexual assault, and compromised academic performance (9). Moreover, binge drinking accounts for the majority of the US economic burden due to alcohol misuse (10). Thus, an improved understanding of the individual-level factors that promote or protect against binge patterns of alcohol use among college students represents a crucial step toward reducing the personal and economic burden of hazardous drinking.

Recent studies have revealed a robust link between attentional ability and resilience against stress-related psychopathology, including AUD (11–14). Resilience refers to a reduction of negative outcomes in the presence of a risk challenge, typically stress or adversity (15), but also occurs in response to heritable risk (16). For example, among individuals undergoing military training, scores on the Barrett Impulsiveness Scale (BIS) mediated the link between depression and stress coping resilience, as measured with the Connor-Davidson Resilience Scale (CD-RISC) (15); a further examination of BIS subscales revealed that this mediation was driven by an attention subscale of the BIS, whereas the mediation was absent for motor impulsivity and non-planning impulsivity subscales. Similarly, among individuals with bipolar disorder, resilience measured with the CD-RISC inversely correlated with BIS total scores and BIS subscales, but the strongest correlation was with the attention subscale (14); these correlations were less robust in a healthy control group. Other evidence for the importance of attention in resilience comes from a study of medical students, which demonstrated that attention-deficit/hyperactivity disorder (ADHD) symptoms of inattention, but not impulsivity, related to lower scores on the CD-RISC, which predicted lower life satisfaction. In treatment-seeking patients with AUD, mindful attention was a mediator between emotion regulation and AUD severity (11), such that greater mindful attention was related to better emotion regulation and lower alcohol use. Furthermore, treating symptoms of ADHD in childhood and adolescence lowers the elevated risk for AUD associated with ADHD (17, 18). However, the association between attention and resilience related to alcohol use has not yet been explicitly tested. If such a link exists, interventions to improve attention in children and adolescents at risk for AUD who present with attentional deficits may reduce hazardous alcohol use by promoting resilience among these individuals.

Among those at greatest risk of developing AUD are individuals with a family history (FH) of AUD. Twin studies estimate that approximately half of the risk for AUD is heritable (19, 20). Thus, FH significantly increases an individual's risk for AUD (21, 22), and this relationship is independent of the increased adversity experienced by children of alcoholic parents (21). We tested the hypothesis that attentional ability confers resilience to hazardous alcohol use in college students with familial risk for AUD. To this end, we examined attention scores between FH positive (FHP) and FH negative (FHN) individuals who engaged in binge alcohol drinking vs. those that did not engage in binge alcohol drinking and found a protective effect of attentional ability among FHP individuals. Using mediation analyses, we examined directional relationships between trait attention, resilience scores, and binge alcohol drinking among college students with or without FH. The results indicated a mediating role for attentional ability in resilience to binge drinking in college students at risk for AUD based on FH.

## Methods

### Subjects

We recruited 464 first-year college students for one of two parallel studies. Study 1 (*n* = 148) consisted of a neuroimaging session as well as online self-report questionnaires. Study 2 (*n* = 316) was designed, largely in response to the COVID-19 pandemic, as an online alternative to the neuroimaging study in which participants completed the same set of self-report questionnaires but did not attend an in-person imaging session. Participants gave written (Study 1) or electronic (Study 2) informed consent in accordance with ethical standards of the UNC Office of Human Research Ethics.

Inclusion criteria for both studies was 18–19 years of age and in their first year in a 4-year undergraduate degree program. Given the strong effects of the college environment on alcohol consumption, and to reduce variability associated with this factor, college attendance at time of study enrollment was an inclusion criterion. The neuroimaging study, Study 1, had additional exclusion criteria: MRI contraindications (i.e., claustrophobia, non-removable metal in the body, pregnancy), left-handedness, psychoactive drug use (including medications), neurological disorders, and psychiatric disorders with the exception of lifetime—but not current—mood or anxiety disorders. Psychiatric disorders were assessed *via* Mini-International Neuropsychiatric Interview (M.I.N.I.) for DSM-IV (23). We excluded for current or lifetime AUD or substance use disorder using DSM-5 criteria. Study 1 subjects also screened negative for alcohol and other drug use (i.e., cocaine, cannabis, opioids, amphetamines, methamphetamine) on the day of the neuroimaging session *via* breathalyzer test (FC-10, Lifeloc Inc., Wheat Ridge, CO) and urine drug screen (Biotechnostix, Inc., Markham, ON), respectively.

### Self-Report Instruments

Subjects completed a computerized battery of self-report questionnaires administered with Research Electronic DATA Capture (REDCap) tools hosted at UNC (24), which included instruments measuring personal and family alcohol use, attention, impulsiveness, resilience, and symptoms of depression.

We administered three items from the Alcohol Use Questionnaire (#10-#12) that were combined to create a “binge drinking score,” as described elsewhere (25).

Timeline followback calendars (26) were administered to collect self-report data on the number of alcoholic drinks consumed on each of the 30 previous days. We calculated the number of binge drinking episodes in 30 days, where a binge drinking was defined as any day in which 5 or more drinks (males) or 4 or more drinks (females) were reported.

AUD among family members was assessed with the Family History Assessment Module (FHAM) (27). We created a binary FH variable based on having a first-degree biological relative (i.e., parent or sibling) or two or more biological second-degree relatives (i.e., grandparents, aunts, and uncles) with AUD. This definition was based on the utility of considering both first-degree and second-degree relatives in AUD research (28, 29).

The CD-RISC is a measure of resilience that contains 10 questions relating to ability to cope with stressors and has been validated for use in college students with histories of stress and trauma (30). Scores range from 10 to 40, with higher scores corresponding with greater stress resilience and mean scores of a community sample of adults being ~32 (31).

Although the BIS was designed as a measure of impulsivity, the BIS factor structure contains a first-order factor that is comprised of items measuring attentional ability (32). Questions contained in the first-order “attention” factor relate to the ability to focus and pay attention, for example, “I don't pay attention,” “I concentrate easily,” and “I am restless at the theater or lectures.” Higher scores correspond with more impaired attentional ability. As our *a priori* hypothesis centered around attention as a mediator of resilience and binge drinking, we used this subscale in our primary analyses. Secondary analyses explored relationships using other first-order and second-order subscales.

Depressive symptoms were measured with the Beck Depression Inventory (33), and a total score was computed to represent current levels of depression.

For descriptive purposes, we calculated the means and standard errors of the means for the above self-report measures, separately by FH status and study. A chi-square test in SAS 9.4 PROC FREQ tested for differences in the distribution of the sexes by FH status and study. Two-way analyses of variance (ANOVAs) in SAS (Cary, NC) PROC ANOVA were performed on BDI scores, CD-RISC scores, and BIS scores to test for main effects of FH status and study. Although we administered the Customary Drinking and Drug Use Record [CDDR, Brown et al. (34)] to assess substance use in addition to alcohol, cannabis use and tobacco use were almost exclusively reported among users of alcohol, with the exception of only two subjects who reported using both cannabis and tobacco but not alcohol. Therefore, statistical analyses could not reliably separate effects of these other substances from alcohol effects, and thus these variables were not included in analyses.

### Attention Scores by Family History and Binge Drinking

To examine evidence for attention as a resilience factor among FHP subjects, we tested for statistical differences in attention between FHP binge drinkers and non-binge drinkers as well as between FHN binge drinkers and non-binge drinkers. Because there is not a specific binge drinking score that clearly delineates binge drinkers from non-binge drinkers due to the nature of the calculation (35), we designated non-binge drinkers as those with a score of 0, representing the first quartile of our sample, who also reported no binge episodes in the previous 30 days based on timeline followback data. Twenty-two subjects with missing timeline followback data were excluded from the non-binge-drinking group due to an inability to confirm their status as non-binge drinkers. We designated binge drinkers as those in the upper quartile of binge scores, which included scores ≥20, regardless of the number of binge episodes in the previous 30 days. Binge drinkers reported an average of 3.44 binge episodes in the previous 30 days (range 0–19).

We conducted a two-way analysis of covariance (ANCOVA, SAS PROC ANOVA) testing factors of FH, binge drinking, and their interaction, covarying for sex and study. Exploratory analyses tested other BIS subscales as dependent variables in similar ANCOVA models without correcting for the multiple tests.

### Correlations Between Resilience, Attention, and Binge Drinking

We assessed correlational relationships between CD-RISC and BDI scores, binge drinking scores, and BIS scores. Spearman rank-order partial correlations were conducted in SAS 9.4 separately by FH status, covarying for study. Statistical significance was determined by calculating 95% confidence intervals from 10,000 bootstrap iterations. We included biological sex as a covariate due to sex differences in binge drinking (Center for Behavioral Health Statistics and Quality 2015) as well as in stress-related psychopathology within attention systems (36). Due to known associations of depressive symptoms with both binge drinking (25) and resilience (13), correlations also covaried for BDI scores to ensure relationships were not driven by depressive symptoms (with the exception of the correlation between CD-RISC and BDI scores).

### Mediation Analyses

Next, we sought to examine causal relationships between attention, resilience, and binge alcohol drinking. Specifically, we used mediation analyses to test whether BIS attention scores mediated the relationship between CD-RISC scores and binge drinking scores, and whether the indirect effect was moderated by FH.

Missing questionnaire data was imputed with the mean value from other subjects from the same study, FH status, and sex. For all variables, missing data represented <4% of values. Mediation analyses were performed using the Multilevel Mediation and Moderation (M3) MATLAB Toolbox (37) in MATLAB 2019b. Due to differences across our study samples (Table 1), we utilized multilevel analyses to merge the samples from Study 1 and Study 2, preserving power to detect variable relationships, while simultaneously allowing separate estimates of means and slopes for the two studies. Significant paths were evaluated by performing 100,000 bootstrap iterations and calculating 95% confidence intervals. Mediation analyses were conducted separately for FHP and FHN groups.

**Table 1:** Sample characteristics of individuals with or without a family history of alcohol use disorder.

	**Family history positive**		**Family history negative**			
	**Study 1 (*n* = 79)**	**Study 2 (*n* = 142)**	**Study 1 (*n* = 69)**	**Study 2 (*n* = 174)**	**FH effect**	**Study effect**
Sex (males/females)	26/53	28/114	28/41	66/108	***p*** **= 0.002**	*p* = 0.19
Connor-Davidson Resilience Scale-10	29.0 (0.71)	27.3 (0.64)	29.4 (0.72)	27.4 (0.50)	*p* = 0.88	***p*** **= 0.007**
Beck depression inventory	8.7 (0.94)	15.5 (1.30)	7.2 (0.96)	11.7 (0.99)	***p*** **= 0.020**	***p*** **< 0.001**
Binge drinking score	12.0 (1.57)	17.6 (1.62)	8.3 (1.25)	13.1 (1.40)	***p*** **= 0.017**	***p*** **= 0.005**
Binge episodes in 30 days	0.51 (0.14)	1.15 (0.26)	0.87 (0.23)	1.00 (0.22)	*p* = 0.13	*p* = 0.85
Barratt Impulsiveness Scale (BIS) total	56.5 (1.07)	61.6 (1.04)	57.6 (1.29)	60.0 (0.81)	*p* = 0.67	***p*** **= 0.001**
BIS 1st-order attention	9.6 (0.29)	10.8 (0.29)	9.9 (0.34)	10.4 (0.22)	*p* = 0.63	***p*** **= 0.006**
BIS 1st-order cognitive instability	6.0 (0.19)	6.7 (0.19)	6.1 (0.21)	6.3 (0.16)	*p* = 0.46	***p*** **= 0.026**
BIS 1st-order motor	13.0 (0.35)	14.1 (0.29)	13.6 (0.39)	13.7 (0.26)	*p* = 0.95	*p* = 0.08
BIS 1st-order perseverance	6.3 (0.15)	6.5 (0.15)	6.5 (0.18)	6.3 (0.10)	*p* = 0.72	*p* = 0.92
BIS 1st-order self-control	11.4 (0.35)	12.5 (0.32)	11.1 (0.39)	12.3 (0.26)	*p* = 0.59	***p*** **= 0.001**
BIS 1st-order cognitive complexity	10.2 (0.26)	11.0 (0.23)	10.2 (0.30)	11.0 (0.18)	*p* = 0.94	***p*** **= 0.002**
BIS 2nd-order attentional	15.6 (0.38)	17.5 (0.43)	16.0 (0.44)	16.7 (0.32)	p = 0.49	***p*** **= 0.003**
BIS 2nd-order motor	19.3 (0.44)	20.6 (0.38)	20.2 (0.50)	20.0 (0.30)	*p* = 0.92	*p* = 0.21
BIS 2nd-order non-planning	21.6 (0.56)	23.6 (0.46)	21.3 (0.61)	23.3 (0.37)	*p* = 0.75	***p*** **< 0.001**

*Frequencies are presented for sex. Means and standard errors of the mean (in parentheses) are presented for continuous measures. Significant p-values are bolded. FH, Family History*.

Prior to each mediation analysis, study variables were converted to rank-ordered values due to non-normal distributions. Next, we used linear regression models to estimate effects of sex and depression on the mediator (BIS attention) and the outcome variable (binge drinking score). These effects were subsequently subtracted from the mediator and outcome variables to adjust our mediation model for the effects of these nuisance variables. This adjustment was conducted separately for each study and FH group.

After testing mediation models for the FHP and FHN groups, we additionally tested the null hypothesis of equal indirect paths for the two models, thereby testing whether the mediation effect was moderated by FH. To test this, we took the difference in the indirect path estimates between the FH groups and the variance estimates for those paths to compute a z-statistic of the difference between models for FHP and FHN (38). A significant effect would indicate that FH moderated the mediating effect of attention on the relationship between resilience and binge drinking.

Secondary exploratory analyses tested the remaining BIS factors as mediators using identical methods as above. The goal of these analyses was to explore whether other aspects of impulsivity, outside of our *a priori* hypothesis regarding attention, mediated resilience effects on binge drinking.

## Results

### Demographic and Psychometric Data

Demographic characteristics of subjects separated by FH status and study are presented in Table 1. FHP subjects demonstrated greater BDI scores and higher binge drinking scores. Study 1 and Study 2 demonstrated differences in CD-RISC scores, BDI scores, binge drinking scores, and BIS total and subscale scores except for those related to motor impulsivity.

### Attention Scores by Family History and Binge Drinking Status

**Figure 1:**
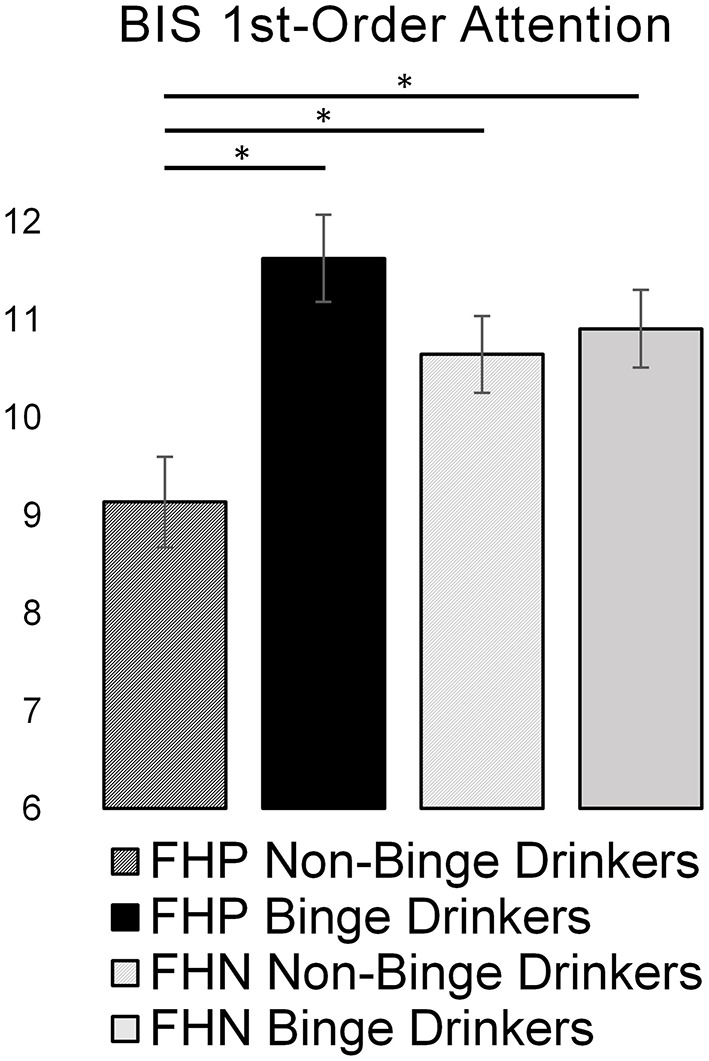
Bar graph representing the effects of family history of alcohol use disorder and binge drinking on Barrett Impulsiveness Scale (BIS) first-order attention scores. Greater attention scores represent more attention problems. Asterisks (*) denote significant group differences (*p* < 0.05). FHP, family history positive; FHN, family history negative.

Scores on the BIS first-order attention subscale by FH and binge drinking group are displayed in Figure 1. Results of a two-way ANCOVA controlling for sex and study indicated a significant interaction of FH and binge drinking [*F*_(1,203)_ = 7.17, *p* = 0.008]. Specifically, non-binge-drinking FHP subjects reported significantly lower attention problems than not only the FHP binge-drinkers, who reported the highest attention problems, but also FHN non-binge drinking subjects, consistent with a marker of resilience to binge drinking.

Exploratory analyses testing other BIS first-order factors in similar ANCOVAs found no other significant interaction effects. Among second-order factors, there was a significant interaction of FH and binge drinking on the attentional subscale [*F*_(1,203)_ = 5.85, *p* = 0.017], mirroring the primary finding (Supplementary Figure 1).

### Correlation Analyses

Correlations between CD-RISC, binge drinking, and BIS scores are presented in Table 2. There was a significant correlation between resilience and attention, similar to previous findings (12, 14), in both FHP and FHN subjects. Additionally, we identified significant correlations between BIS attention scores and binge drinking scores, but only among FHP subjects. Similarly, significant correlations between CD-RISC scores and BIS total and subscale scores were more robust among FHP. On the other hand, the correlation between resilience and depression, replicating previous work (13, 14), was strongest in FHN subjects.

**Table 2:** Correlations between Connor-Davidson Resilience Scale (CD-RISC) scores and clinical measures in individuals with or without a family history of alcohol use disorder.

	**Family history positive**	**Family history negative**
	**ρ (95% CI)**	**ρ (95% CI)**
Beck depression inventory	**−0.21 (−0.35, −0.06)**	**−0.43 (−0.54, −0.30)**
Binge drinking score	**−0.15 (−0.29, −0.01)**	0.01 (−0.11, 0.14)
Barratt Impulsiveness Scale (BIS) total	**−0.33 (−0.46, −0.19)**	**−0.25 (−0.38, −0.12)**
BIS 1st-order attention	**−0.40 (−0.52, −0.27)**	**−0.18 (−0.31, −0.05)**
BIS 1st-order cognitive instability	0.01 (−0.13, 0.15)	−0.13 (−0.31, 0.00)
BIS 1st-order motor	0.04 (−0.11, 0.17)	−0.02 (−0.16, 0.11)
BIS 1st-order perseverance	**−0.29 (−0.42, −0.16)**	**−0.20 (−0.32, −0.07)**
BIS 1st-order self-control	**−0.37 (−0.49, −0.24)**	**−0.24 (−0.36, −0.10)**
BIS 1st-order cognitive complexity	**−0.36 (−0.49, −0.22)**	**−0.34 (−0.45, −0.22)**
BIS 2nd-order attentional	**−0.26 (−0.39, −0.13)**	**−0.20 (−0.33, −0.06)**
BIS 2nd-order motor	−0.07 (−0.21, 0.07)	−0.09 (−0.22, 0.05)
BIS 2nd-order non-planning	**−0.42 (−0.54, −0.28)**	**−0.33 (−0.45, −0.20)**

*Spearman's correlation coefficients (ρ) and 95% confidence intervals (CI) are presented. Significant correlations are bolded*.

### Mediation Analyses

**Figure 2:**
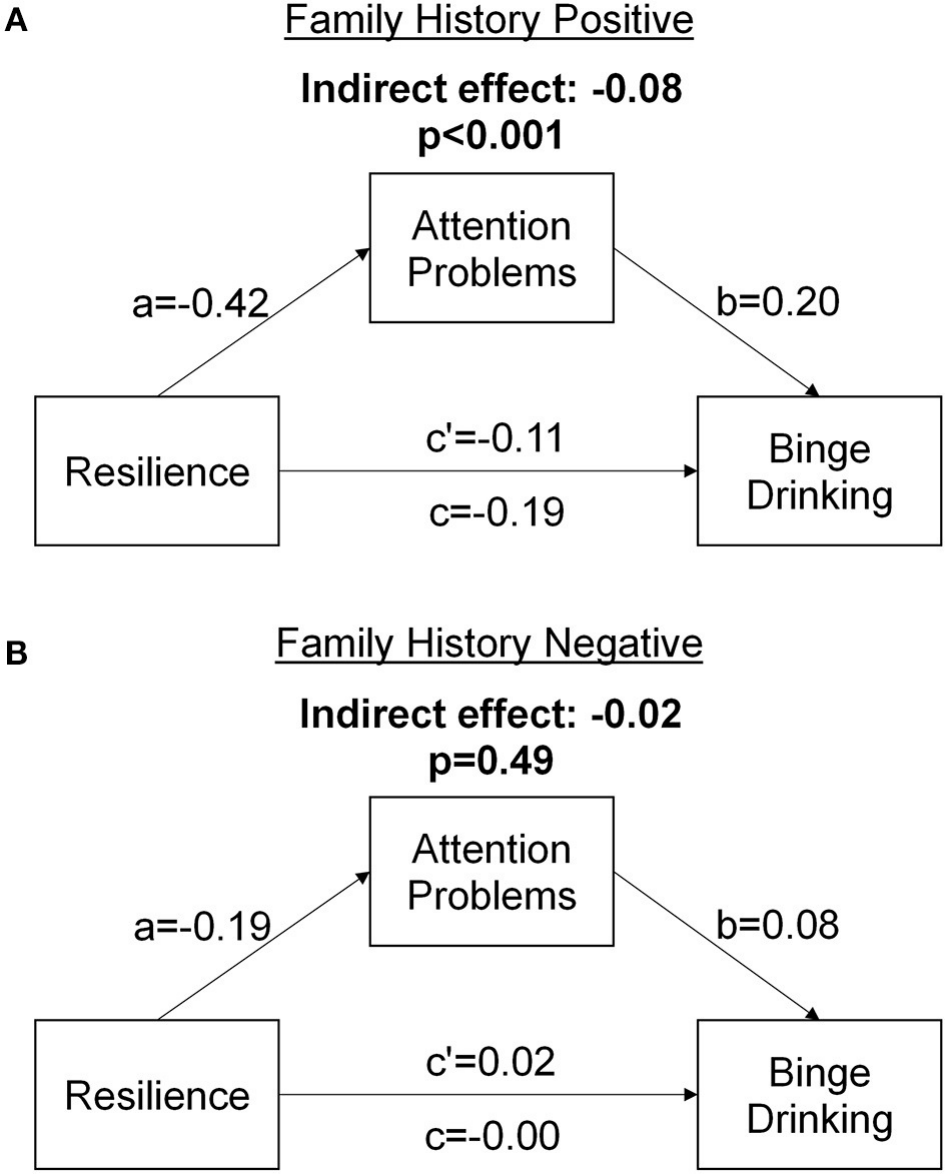
Mediation models demonstrating the mediating effect of Barrett Impulsiveness Scale (BIS) attention scores on the relationship between Connor-Davidson Resilience Scale (CD-RISC) scores and binge drinking scores for **(A)** individuals with a family history of alcohol use disorder and **(B)** individuals with no a family history of alcohol use disorder.

Models of the mediating effects of BIS first-order attention scores on the relationship between CD-RISC and binge drinking are presented in Figure 2. The direct path between CD-RISC scores and binge drinking score was significant for FHP, but not FHN, subjects. There were significant *a* and *b* paths in both groups, indicating an indirect effect of resilience on attention and an effect of attention on binge drinking. However, the total indirect effect (*a*×*b*) of BIS Attention scores was stronger for FHP than for FHN (*z* = −3.05, *p* = 0.002).

Mediation effects for all BIS subscales are presented in Supplementary Table 1. Of note, there was a sizable and significant mediation by the non-planning second-order subscale of the BIS, suggesting reductions in this form of impulsiveness may also promote stress coping resilience to reduce binge drinking.

## Discussion

In this study, we tested a hypothesis that enhanced attentional ability promotes resilience to binge alcohol drinking in at-risk emerging adults. The findings supported a role for higher trait attention in reducing binge drinking among first-year college students with familial risk for AUD. We further demonstrated that attention scores mediated the relationship between a measure of resilient coping (i.e., CD-RISC) and binge drinking among first-year college students and that this relationship was stronger for FHP than FHN. The results suggest that even though attention may only be weakly correlated with alcohol use in the general population, it may actually play a key role in promoting resilience to binge drinking among FHP individuals.

As expected, FHP subjects had higher binge drinking scores, consistent with previous research (39). In addition, the relationship between resilience and binge drinking scores was only present among FHP, and the indirect path through attention scores was stronger for FHP than FHN. Conversely, resilience was more strongly related to depression scores in FHN subjects. This dissociation suggests that the effects of stress resilience in reducing psychiatric symptoms may depend on having a certain underlying susceptibility. In our samples, FHP did not demonstrate increased impulsivity and attention problems as measured by the BIS (Table 1). However, FH has previously been associated with executive function deficits related to impulsivity and attention (40–42), and the current study indicated that individual variation in these measures predicted binge drinking. It is possible that a less resilient sample or more sensitive measures would have revealed FH effects on impulsivity and attention.

Much of the research on resilience implicates a role for dopamine signaling. For example, preclinical models of resilience have identified associations with stable firing of ventral tegmental area dopamine neurons projecting to the nucleus accumbens (43), increased D2 receptor mRNA expression (44), and activation of indirect pathway D2-expressing medium spiny neurons in the nucleus accumbens (45). Resilience is also related to dopamine genes in humans exposed to stress, including a polymorphism of DRD4, increased expression of dopamine receptor genes, and decreased expression of genes encoding dopamine metabolizing enzymes (46, 47). Of direct relevance to AUD, increased striatal D2 receptor availability in FH individuals without AUD has been proposed as a marker of resilience (48). Furthermore, methylphenidate-induced increases in extracellular dopamine in the ventral striatum and medial prefrontal cortex relate to improved symptoms of inattention (49), and methylphenidate reduces risk for addiction in individuals with ADHD (17, 18). The mesolimbic dopamine system influences both emotion regulation and executive functioning (50), and the developmental functional neuroanatomy of these functions is closely linked (51), providing a possible conceptual link between dopamine, stress resilience, and attentional ability.

There are several possible behavioral mechanisms by which attention may promote resilience in at-risk individuals. First, attentional ability may attenuate alcohol misuse through its effects on alcohol-related attentional bias. Attentional bias, which describes an exaggerated attention to salient stimuli, is a predictor of craving and substance use outcomes. Attention problems have previously been associated with increased attentional bias among substance misusers, suggesting a close relationship between the construct of attention and this marker of addiction-related outcomes. Secondly, attention is closely related to mindfulness, which promotes resilience to stress. Indeed, mindful attention is associated with improved emotion regulation in both individuals with AUD (11) and those without (52) and may also reduce drinking (53, 54). Similarly, stress responses influence prefrontal functioning, and heightened stress responding may reflect a failure to exert top-down attentional ability over stress systems and attention to threat (55). Thus, the relationship between resilience to stressors (i.e., CD-RISC scores) and attentional ability may reflect variations in prefrontal-limbic circuitry. Finally, it is possible that attentional ability does not causally reduce drinking among FHP individuals, but may rather represent a behavioral marker of resilience due to its correlation with causal brain mechanisms. Further tests of these hypothesis will be required to determine the role of attention in AUD resilience.

Addiction is a complex and heterogeneous disorder (56, 57), and thus interventions targeting specific deficits in subgroups of individuals represents a logical treatment strategy (58). Preventive measures targeting highly vulnerable populations may be most effective (59). The current study findings suggest that treatments targeting attention processes in individuals with heritable risk for AUD could reduce hazardous alcohol use. A number of treatments for inattentive symptoms have been developed. Pharmacological treatments to reduce inattention associated with ADHD includes psychostimulants such as methylphenidate or non-stimulant drugs like atomoxetine (60). Efficacious non-pharmacological treatments include neuro-feedback using EEG, which demonstrates large, sustained effects on inattentive symptoms (61). Computer-based attention training has also been successfully used to promote attentional ability in various populations (62–64). Finally there is evidence that mindfulness training can improve attentional ability (65–67) including reductions in attentional bias (66). Medications targeting attention processes, computer-based cognitive training, and mindfulness have been tested as potential treatments for substance use disorders, with limited success (68–70). However, the efficacy of such interventions among emerging adults with heritable risk for AUD remains to be determined.

Secondary analyses (Supplementary Materials) also revealed a significant and noteworthy role of non-planning impulsiveness in mediating effects of CDRISC scores on binge drinking. The non-planning subscale of the BIS reflects a lack of focus on the future and correlates with discounting of delayed rewards (71). Impulsivity represents a well-studied risk factor for addiction (72, 73). Given its strong association with both CDRISC scores and binge patterns of alcohol drinking, non-planning impulsiveness may represent another useful target for reducing hazardous drinking related to poor stress coping among FHP individuals. Future work should consider the roles of future-oriented thinking in promoting resilience to hazardous alcohol use among at-risk individuals.

### Limitations

Several limitations of the study should be considered. This study was conducted only in first-year undergraduate students attending 4-year colleges and thus the results may not necessarily translate to adolescents, older populations, or 18–19-year-olds in other academic or non-academic environments. Because analyses did not control for other substance use, it is possible that reported relationships are not specific to alcohol use. Additionally, this study relied on self-report measures of substance use, attention, and resilience. The cross-sectional nature of this study is another limitation, and future studies are needed to determine whether the association of attentional ability with resilience and binge drinking represents a causal mechanism. It is also possible that many of the non-binge drinking subjects will develop AUD later in life. The utility of attentional ability as a predictor of future AUD remains to be determined. Additionally, a large proportion of our sample was female, and the study was not powered for testing sex differences, so it is possible that effects differ between males and females. Increased severity of clinical symptoms in the online sample (Study 2) was likely driven by the relaxed exclusion criteria, which was intended to provide improved real-world translation. However, differences in recruitment methods, geographical regions, and the pandemic may also have contributed to sample differences. Future studies are needed to identify the neural circuits that mediate the relationships between attentional ability, resilience, and binge drinking.

### Conclusions

This study revealed a novel link between attention and resilience to binge alcohol use among individuals at risk for AUD. Reducing hazardous alcohol use on college campuses remains an important goal of intervention research (52). The current findings suggest approaches targeting attention in college students with familial risk for AUD have the potential to reduce the immediate and future burden of binge drinking in this high-risk group.

## Data Availability Statement

The raw data supporting the conclusions of this article will be made available by the authors, without undue reservation.

## Ethics Statement

The studies involving human participants were reviewed and approved by Institutional Review Board at the University of North Carolina at Chapel Hill. The patients/participants provided their written informed consent to participate in this study.

## Author Contributions

AE was responsible for study design, data acquisition, statistical analyses, and drafting of the manuscript. JA was responsible for data acquisition and revisions. MY and FK was responsible for data acquisition and manuscript revisions. QL was responsible for calculations of timeline followback data and manuscript revisions. QL was responsible for calculations of timeline followback data and manuscript revisions. CB advised on study design and provided manuscript revisions. All authors contributed to the article and approved the submitted version.

## Conflict of Interest

The authors declare that the research was conducted in the absence of any commercial or financial relationships that could be construed as a potential conflict of interest.
