# Supplementary material for: High Trait Attention Promotes Resilience and Reduces Binge Drinking Among College Students With a Family History of Alcohol Use Disorder
Source: Front Psychiatry. 2021 May 13;12:672863. doi: 10.3389/fpsyt.2021.672863 (PMC8155514; doi:10.3389/fpsyt.2021.672863)
Supplement: Supplementary file 2 [file Data_Sheet_1.PDF]

Supplementary Material

1. Supplementary Figures

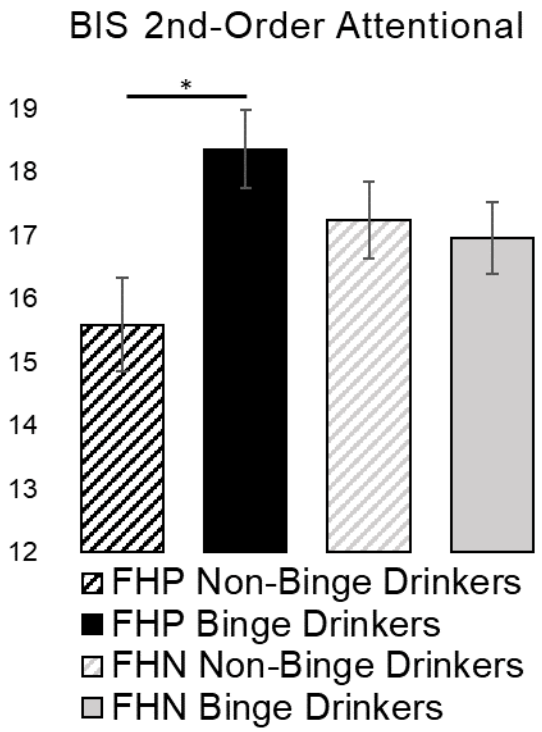

Supplementary Figure 1. Bar graph representing the effects of family history of alcohol use disorder and binge drinking on Barrett Impulsiveness Scale (BIS) second-order attention scores. Greater attention scores represent more attention problems. Asterisks (*) denote significant group differences ($p<0.05$). FHP, family history positive; FHN, family history negative.
